# High-Risk Neuroblastoma Stage 4 (NBS4): Developing a Medicinal Chemistry Multi-Target Drug Approach

**DOI:** 10.3390/molecules30102211

**Published:** 2025-05-19

**Authors:** Amgad Gerges, Una Canning

**Affiliations:** 1 School of Human Sciences, London Metropolitan University, 166-220 Holloway Rd., London N7 8DB, UK; 2 Independent Researcher, London SE4 1JT, UK; u.canning1@gmail.com

**Keywords:** neuroblastoma stage 4 (NBS4), receptors, compounds, multi-target drugs, docking, cross-docking, binding interaction

## Abstract

Childhood neuroblastoma (NB) is a malignant tumour that is a member of a class of embryonic tumours that have their origins in sympathoadrenal progenitor cells. There are five stages in the clinical NB staging system: 1, 2A, 2B, 3, 4S, and 4. For those diagnosed with stage 4 neuroblastoma (NBS4), the treatment options are limited with a survival rate of between 40 and 50%. Since 1975, more than 15 targets have been identified in the search for a treatment for high-risk NBS4. This article is concerned with the search for a multi-target drug treatment for high-risk NBS4 and focuses on four possible treatment targets that research has identified as having a role in the development of NBS4 and includes the inhibitors Histone Deacetylase (HDAC), Bromodomain (BRD), Hedgehog (HH), and Tropomyosin Kinase (TRK). Computer-aided drug design and molecular modelling have greatly assisted drug discovery in medicinal chemistry. Computational methods such as molecular docking, homology modelling, molecular dynamics, and quantitative structure–activity relationships (QSAR) are frequently used as part of the process for finding new therapeutic drug targets. Relying on these techniques, the authors describe a medicinal chemistry strategy that successfully identified eight compounds (inhibitors) that were thought to be potential inhibitors for each of the four targets listed above. Results revealed that all four targets BRD, HDAC, HH and TRK receptors binding sites share similar amino acid sequencing that ranges from 80 to 100%, offering the possibility of further testing for multi-target drug use. Two additional targets were also tested as part of this work, Retinoic Acid (RA) and c-Src (Csk), which showed similarity (of the binding pocket) across their receptors of 80–100% but lower than 80% for the other four targets. The work for these two targets is the subject of a paper currently in progress.

## 1. Introduction

**Figure 1:**
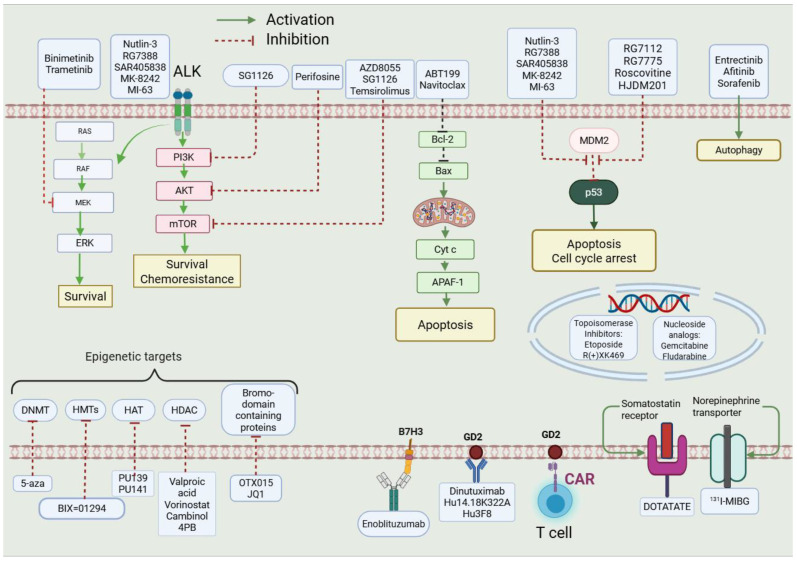
Targeted therapy in NB. Reproduced from Ref. [5], copyright 2021, recreated with BioRender.com (accessed on 10 November 2023).

Childhood neuroblastoma (NB) is a malignant tumour that is a member of a class of embryonic tumours that have their origins in sympathoadrenal progenitor cells [1]. There are five stages in the clinical NB staging system: 1, 2A, 2B, 3, 4S, and 4 [1]. For those diagnosed with stage 4 neuroblastoma (NBS4) the treatment options are limited with a survival rate of between 40 and 50% [2]. This article is concerned with the search for a multi-target drug treatment for high-risk NBS4 and focuses on four possible treatment targets. Since 1975, more than 15 targets have been identified in the search for a treatment for high-risk NBS4 [3]. Six targets were originally selected and of these, on the basis of the most promising research (Figure 1) [4] Four demonstrated amino acid sequence similarity of 80–100% in the receptors binding sites and are the focus of this paper. The other two targets showed similarity across their receptors of 80–100% but lower than 80% for the other four targets.

The four targets were selected because of promising results in previous research for NBS4. The four have been investigated separately for the role they play in the development of NB and include: Histone Deacetylase (HDAC), Bromodomain (BRD), Hedgehog (HH), and Tropomyosin Kinase (TRK). Of the four BRD, HH and HDAC are epigenetics, a term used to describe the study of heritable traits or a stable change in cell function, that happen without changes to the DNA sequence. In the case of cancers, it is nearly impossible to reverse genetic alterations, whereas epigenetic changes “can dynamically respond to signals from the physical, biological and social environment” [6]. Other targets investigated for NBS4 include Tyrosine Kinases such as MYCN that is involved in gene amplification in NB.

Current treatment for NBS4 involves immunotherapy combined with anti-cancer drugs [7]. With therapeutic agents meeting with little success in treating NBS4, the search for a new target remains urgent [4]. Currently, treatment agents focus on a one-drug-one-target approach and/or combination therapy, which has had little success in improving survival rates. An alternative approach is to develop a multi-target drug that interacts with multiple targets with high efficacy to change the disease network. Further perceived benefits to developing a multi-target drug offer the possibility of making “cocktail therapies” or drug combinations redundant [8], leading to less pharmacokinetic and safety profile testing, as the risk of drug–drug interactions would be reduced [9]. Since it is uncommon for multiple targets to mutate simultaneously in different pathways or at various locations within a single cascade pathway, multi-target drugs may also avoid drug resistance brought on by single-target mutations or changes in expression [10].

This article presents a developmental, multi-targeted drug design approach using computational methods available to medicinal chemistry. Computer-aided drug design and molecular modelling have greatly assisted drug development within the field of medicinal chemistry [11]. Computational methods such as molecular docking, homology modelling, molecular dynamics, and quantitative structure–activity relationships (QSAR) are frequently used as part of the process of finding new therapeutic drug targets [12]. Using these methods, eight compounds (inhibitors) were identified as possible inhibitors for four targets. Results revealed that all four targets BRD, HDAC, HH, and TRK share similar amino acid sequencing in the binding site that ranges from 80 to 100%, offering the possibility of further testing for their suitability for multi-target drug use. Two additional targets were also tested as part of this work, Retinoic Acid (RA) signalling pathway and c-Src kinase (Csk), which showed similarity (of the binding pocket) across their receptors of 80–100% but were lower that 80% for the other four targets.

### 1.1. Reasoning Behind Selected Targets

The four targets were selected due to their downregulation of NBS4:

(1) Histone Deacetylase (HDAC)

The Histone Deacetylase (HDAC) family comprises 18 enzymes divided into four classes (I, II, III, and IV) according to their enzymatic activities, subcellular localisation, and homology to yeast HDCA [13]. In the case of HDAC 8 (class I), it was found to be downregulated in NBS4 (Figure 1) [14]. Two compounds, 8b and 20a [15], and two receptors, 2V5X and 2V5W [16], were selected for this target (see Table 1).

(2) Bromodomain (BRD)

Early in the 1990s, the Brahma gene of Drosophila Melanogaster was found to contain a family of evolutionarily conserved motifs known as Bromodomains (BRD) [17]. Numerous studies have been conducted on the Bromodomain and extra terminal (BET) family. It consists of BRDT, BRD2, BRD3, and BRD4, all of which are widely expressed, with the exception of BRDT, which is only expressed in the testis [18]. BRDs bind histone tail acetylated lysines, recognizing the acetyl group is essential for recruiting additional chromatin factors and transcriptional machinery, which controls gene transcription [18]. The BET family also functions as a cell cycle regulator with BRD4 regulating the expression of genes necessary for the transition from the M to early G1 phase [18]. Research found that the compound JQ1, a BRD inhibitor, upregulated p27 and the proapoptotic gene BIM while suppressing MYC expression, resulting in G1 cell cycle arrest [19,20]. Studies on the Bromodomain inhibitor BET762 in vivo have also shown that it has anti-cancer properties [21]. Two compounds, JQ1 and BET762, and two receptors, 4BJX and 5UY9 [22], were selected for this target (see Table 1).

(3) Hedgehog (HH)

**Figure 2:**
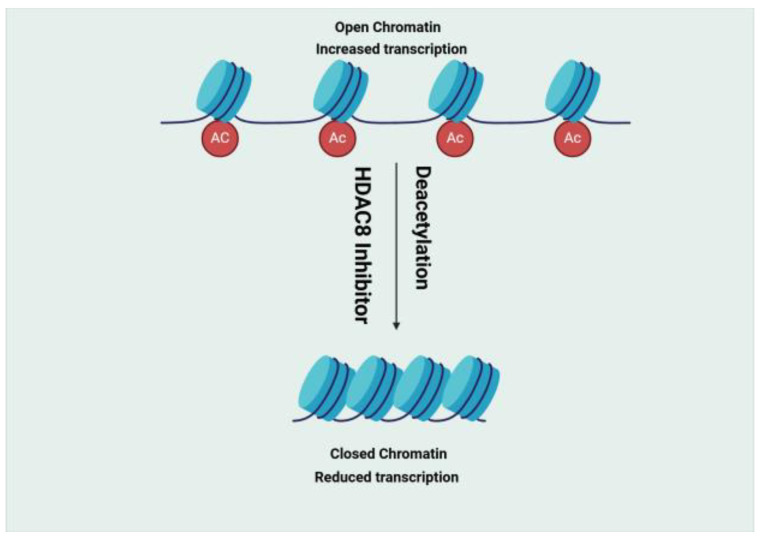
Deacetylation by histone deacetylase 8 (HDAC8), along with the inhibition of HDAC8 by the HDAC8 inhibitor. Created in BioRender. Gerges, A. (2025), https://BioRender.com/r11c559.

Hedgehog Inhibitors (HHIs) have become a promising new target for cancer therapy [23]. The signalling pathway identified in 1980 [24] was a crucial regulator of growth, patterning formation, and cell migration during embryonic development [25]. The components of the HH signalling pathway are involved in signalling to the transcription factors [26]. One study found that signalling deregulation was observed with Gorlin syndrome and cancers (Figure 2) [27]. Two compounds, BMS-833923 [28] and Vismodegib [29], and two receptors, 5L7I [30] and 3N1P [31], were selected for this target (see Table 1).

(4) Tropomyosin Receptor Kinase (TRK)

**Figure 3:**
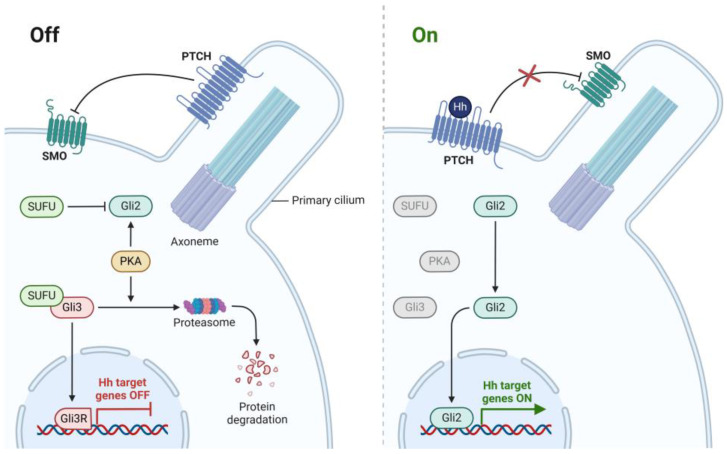
Hedgehog pathway activation in cancer. Created in BioRender. Gerges, A. (2025), https://BioRender.com/w90s818.

The neurotrophin family of peptide hormones activates three related tyrosine kinases known as tropomyosin receptor kinases (TRK) [32] that along with various forms of cancer, are also essential in neurodegenerative diseases. TRK inhibitor development to target cancers driven by NTRK fusion has gained attention in the past ten years (Figure 3) [33].

TRK activation results in the autophosphorylation of an intracellular tyrosine residue [34]. This phosphorylation is a crucial step in activating the TRK receptor and initiating downstream signalling cascades [35]. Two compounds, GW441759 and Compound **10** [36], and two receptors 4AT3 [36] and 3V5Q [36], were selected for this target.

**Table 1:** Selected lead compounds and receptors for each target type by literature review.

Target	Lead Compound 1	Lead Compound 2	Protein/Receptor
1- Histone Deacetylase 8 Inhibitors	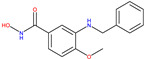	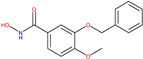	2V5X and 2V5W [16]
	8b [15]	20a [15]	
2- Bromodomain Inhibitors	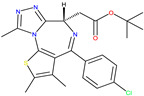 JQ1 [18,20]	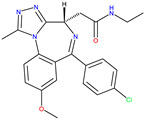 BET762 [18]	4BJX [18] and 5UY9 [22]
3- HH inhibitors	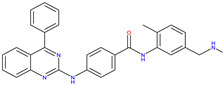 BMS-833923 [28]	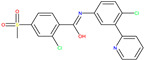 Vismodegib [29]	5L7I [30] and 3N1P [31]
4- Tropomyosin Receptor Kinase Inhibitors	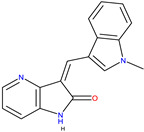 GW441759 [36]	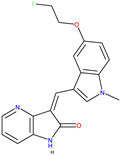 Compound **10** [36]	4AT3 [36] and 3V5Q [36]

### 1.2. Medicinal Chemistry Approaches to Drug Design

Rational multi-target drug design strategies in recent years have varied from pharmacophore combination, screening [37], and similar scaffold structure [38]. Each strategy has advantages and disadvantages, along with varying challenges. The purpose of this work is to suggest a possible modified approach. In the search for a multi-target drug for NBS4 using advanced medicinal chemistry software, this approach assesses the possibility of different targets by assessing receptor similarities (in the binding pocket) and the selection of two lead compounds for each of the four target (eight compounds in total) that have demonstrated an inhibitory effect on NBS4 cells, to form the basis of a search for a multi-target drug.

**Figure 4:**
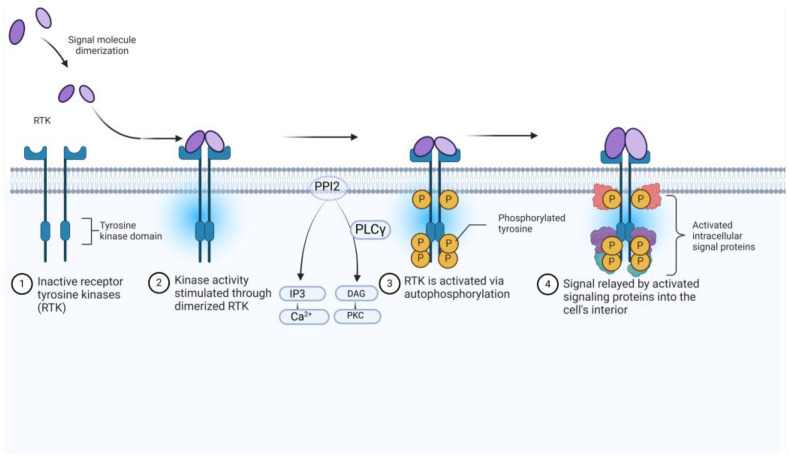
Receptor Tyrosine Kinases (RTKs). Created in https://BioRender.com by Ruslan Medzhtov, Akiko Iwasaki, and Jung-Hee Lee.

Having identified four targets that share similar amino-acid sequencing, two receptors from each target were selected (see Table 1) and a Protein Aligner tool from Samson was used to check for their suitability, with results reporting 80–100% similarity for the four targets (Figure 4). Protein Aligner checks for amino acid sequence similarity between receptors with high similarity between receptors indicating their suitability for use in cross-docking.

The selection of ligands was based on literature research in relation to NBS4. Two compounds were selected for each target (see Table 1), representing a total of eight compounds.

For the purposes of this work docking involves docking a compound to a receptor known for that target (i.e., BRD) whereas cross-docking is docking the same compound to a receptor that belongs to a different target (i.e., TRK) and vice versa. The aim of cross-docking is to explore the suitability of the selected compound for use across different targets to determine its suitability as a multi-target drug. This is a process that involves selecting known lead compounds to produce hitlists of compounds and was performed using BROOD [39] as part of the OpenEye suite.

**Figure 5:**
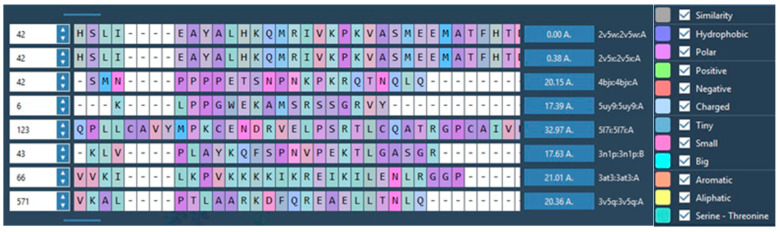
Print screen from Protein Aligner of 2V5W with all the selected receptors from the four targets. The similarity (in grey) was obtained using Protein Aligner by Samson [47]. Zero indicates 100% similarity, and 100% means no similarity. Figure 5 shows that the similarities (in the binding pocket) between the selected proteins range from 80% to 100%.

**Figure 6:**
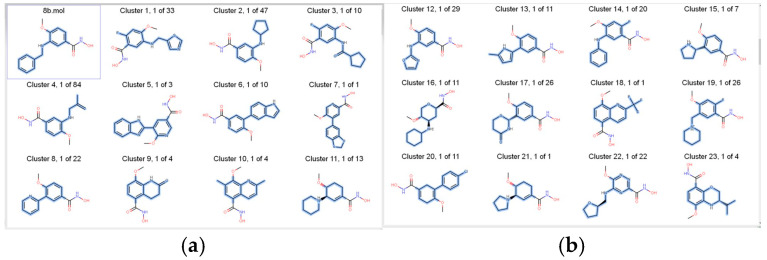
Print screen of BROOD (Shape and Colour) outcome when using 8b as a lead compound. The figure on the left (**a**) shows the first 11 compounds made from 8b, with the figure on the right (**b**) showing another 12.

**Figure 7:**
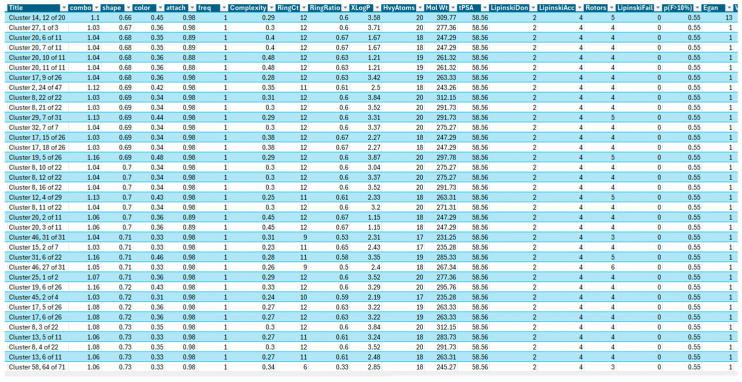
Print screen is taken from the BROOD hitlist file, which contains the results in CSV format; it does not represent the entire outcome. The figure also shows some of the results of the BROOD parameters.

**Figure 8:**
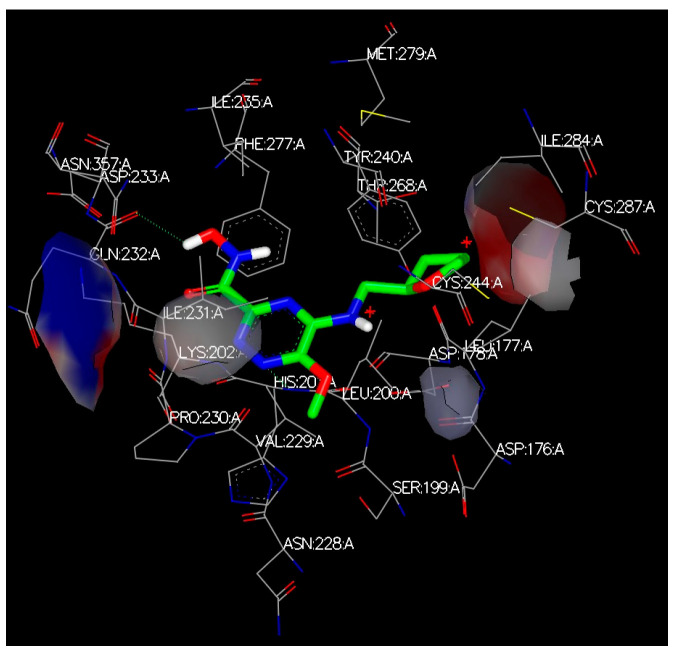
A print screen from FRED shows the docking result of 2V5W (HDAC receptor) with cluster 22 ,1 of 1.

**Figure 9:**
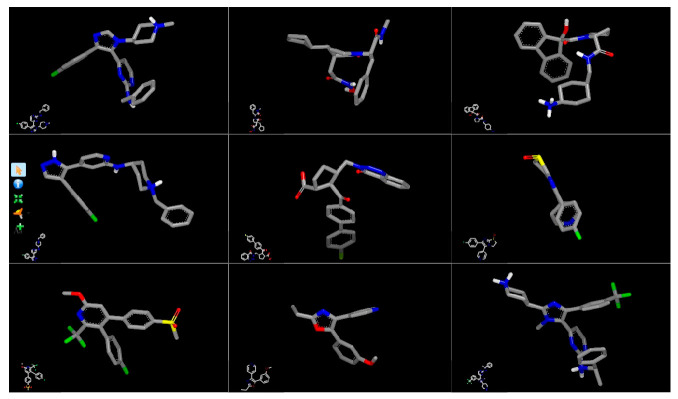
A print screen from ROCS showing the outcome BET762 (BRD inhibitor).

**Figure 10:**
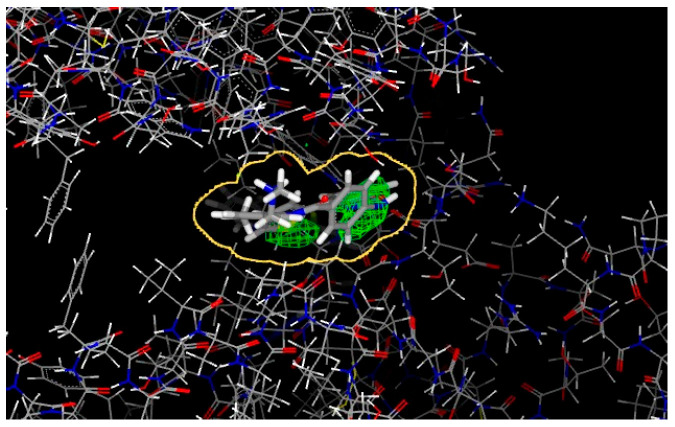
A print screen from AFITT showing an example of HHI clusters with protein 5L7I. Also, some of the fitting results.

**Figure 11:**
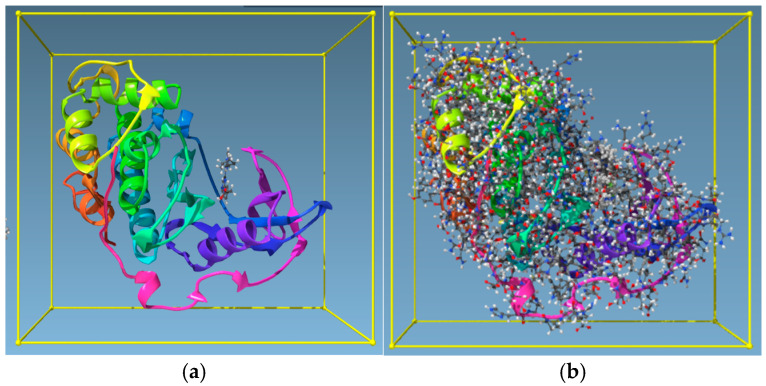
A screenshot from Samson (AutoDock Vina Extended): TRKI (clusters) with 4AT3 before docking (**a**) and after docking (**b**).

**Figure 12:**
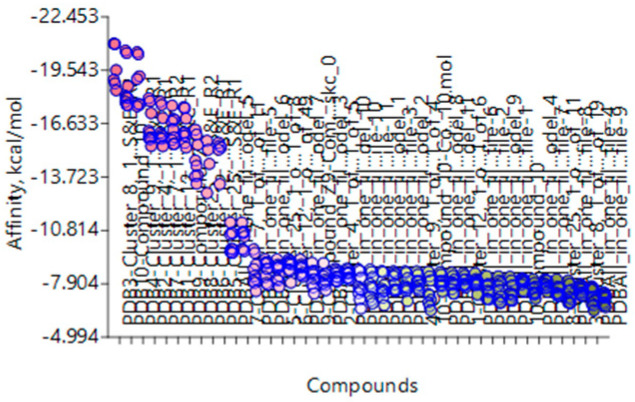
This is a screenshot from AutoDock Vina Extended showing the docking ranking of the TRK receptor 4AT3 with TRKI-selected clusters. The programme uses nine poses for each cluster.

**Figure 13:**
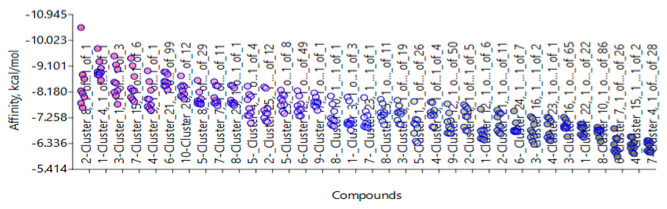
A Screenshot from AutoDock Vina Extended in Samson showing an example of cross-docking. HDAC Receptor 2V5W with BRD, HH, and TRK of multi-target inhibitors.

Several BROOD rounds were completed on the 2 lead compounds for each target to produce hitlists (Figure 5) using “Shape and Colour” and “Shape and Electrostatics” (see Table 2). On completion, BROOD ranked each of the hitlists according to BROOD hitlist parameters (Figure 6), the top 25 of each hitlist was selected, and the best 8–10 compounds of those 25 were selected (see Table 3) to run on OEDocking [40] using FRED (Figure 7), ROCS [41] (Figure 8), AFITT [42] (Figure 9) (see Table 4), and VIDA [43]. In addition, the Samson docking suite, including AutoDock Vina Extended (Figure 10, Figure 11 and Figure 12)) and Fitted (Molecular Forecaster) [44], with another docking programme, Molegro [45], acted as confirmation for both the docking and cross-docking procedures (see Table 5). BIOVIA Discovery Studio was also used for visualization (Figure 13) [46]. From this process, 8 to 10 compounds were selected for each target (see Table 6), all of which showed improved parameters compared to the original lead compounds. The compounds from each individual target were then cross-docked with the receptors from the other three using AFITT. From the 35 compounds run only 8 showed potential suitability for multi-target use (two from each list) (see Table 6).

Selected clusters from all targets were also docked with the Samson Suite, including AutoDock Vina Extended (V 5.1.3) and Fitted by Molecular Forecaster (V 1.7.2).

To check and compare the compounds (clusters) for toxicity and mutagenicity, Toxicity Estimation Software Tools (TEST) (Version 5.1.2) were used to provide the prediction mechanisms of the toxic action of the clusters [48]. The results from TEST showed some similarity with the original lead compounds (Table 7) which also provided their suitability for use. Further work was performed to explore possible synthesis routes for each of the eight compounds using the retrosynthesis programme Spaya [49].

## 2. Results

### 2.1. Medicinal Chemistry Results

Table 1 below contains the selected targets, protein/receptors (Protein Data Bank), and the identified lead compounds.

The next stage was to compare the similarity of the proteins (receptors) binding sites for all four targets. This was achieved using the Samson Protein Aligner tool, showing that the similarities between the selected proteins ranged from 80% to 100% (Figure 4).

By selecting an active group in the lead compound, the programme can produce a hitlist using shape and colour and shape and electrostatics. Table 2 shows how many rounds were performed for each lead compound according to target. The top 25 compounds from the hitlists were selected for the docking studies from each round. Figure 5 shows some of the compounds.

BROOD [50] hitlist parameters (see Figure 6) include the following:

(1) **AroRingCt:** Number of aromatic rings in the molecule;
(2) **ClusterID/IdeaGroup:** ClusterID of the molecule;
(3) **Colour:** The replacement fragment’s colour Tanimoto score in comparison to the query fragment;
(4) **Combo:** Tanimoto combo score for the replacement fragment’s shape and colour in comparison to the query fragment;
(5) **Egan:** The Boolean indicates if the molecule satisfies the Egan bioavailability model;
(6) **Fragment:** SMILES string of the replacement fragment;
(7) **Freq:** The replacement fragment’s frequency;
(8) **fsp3C:** The molecule’s fraction of sp3 hybridized carbon atoms;
(9) **HvyAtoms:** Number of heavy atoms in the molecule;
(10) **LipinskiDon:** Number of Lipinski donors in the molecule;
(11) **LipinkskiAcc:** Number of Lipinski acceptors in the molecule;
(12) **LipinskiFail:** Boolean specifying whether the molecule fails Lipinski’s rule of five;
(13) **Local strain:** Calculated local strain of the molecule;
(14) **Molecular TanimotoCombo:** Shape + colour Tanimoto combo score of the molecule against the query molecule;
(15) **MolWt:** Molecular weight of the molecule;
(16) **p (active):** Belief score of the molecule;
(17) **RingCt:** Number of ring atoms;
(18) **RingRatio:** Ratio of the number of ring atoms to the total number of heavy atoms;
(19) **Rotors:** Number of rotatable bonds in the molecule;
(20) **Shape:** Compare the replacement fragment’s Shape Tanimoto score to that of the query fragment;
(21) **Source Mols:** SMILES strings of the molecules the replacement fragment is part of;
(22) **Source Mol Labels:** Labels of the molecules the replacement fragment is part of;
(23) **tPSA:** Calculated topological polar surface area of the molecule;
(24) **Veber:** Boolean specifying whether the molecule passes the Veber bioavailability model;
(25) **XlogP:** Calculated LogP of the molecule [50].

The next stage was to dock each hitlist with their relevant receptors; Figure 7 shows one of the docking outcomes using FRED (OpenEye suite) at the top of the list cluster 22, 1 of 1.

Having completed all the docking using FRED, Molegro, AutoDock Vina, and Fitted only the top compounds for each target were selected (range 8 to 10 see Table 3) to be run for validation on ROCS a programme that scores and aligns a database of molecules with a query. The score assigns a number to molecules according to their likelihood of having biological characteristics in common with the query molecule (Figure 8).

Another crystallographic tool used from the OpenEye suite is AFITT (Figure 9). AFITT creates a new combined forcefield that fits small molecules into crystallographic density while preserving superior chemistry by combining the shape and MMFF technologies of OpenEye. In order to verify the refinement, it also offers an interface to external refinement programmes, such as real space correlation coefficient calculation (RSCC) and interactive Ramachandran plots.

All possible clusters (Table 3) were docked with all docking tools: FRED, AFITT, AutoDock Vina Extended, Molegro, and Fitted. Table 4 provides an example.

Table 5 shows the docking of selected clusters from each target with more than one receptor of the same target.

**Figure 14:**
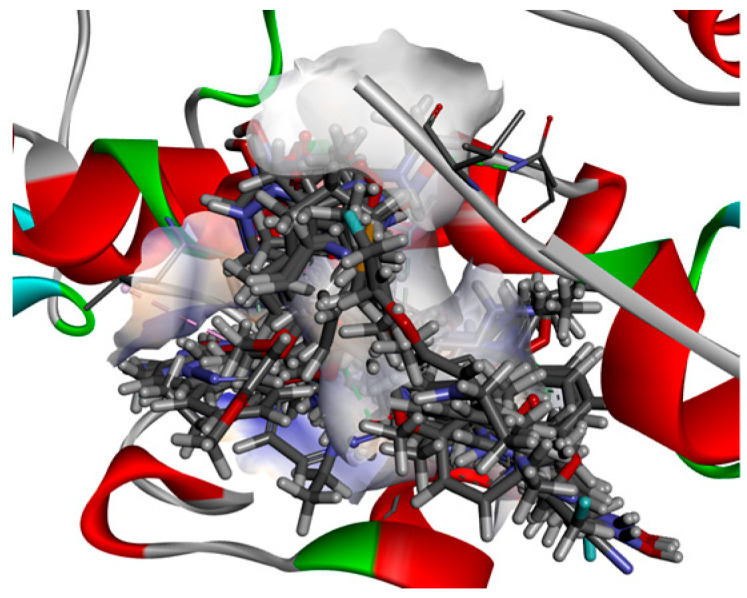
A screenshot showing the docking. HDAC Receptor 2V5X with BRD, HH, and TRK inhibitors using Samson Fitted in BIOVIA Studio Discovery Visualizer [46].

The next step was cross-docking, which involved docking receptors with various cluster types and comparing the results. Figure 14 shows some of the results from the cross-docking of each target.

Having completed all cross-docking for the receptors and the selected clusters, eight compounds were identified as possible multi-target compounds (see Table 6 below).

### 2.2. Retrosynthesis Results Using Spaya

(1) **Synthesis of Cluster 22, 1 of 22 R1 S&C** [49]

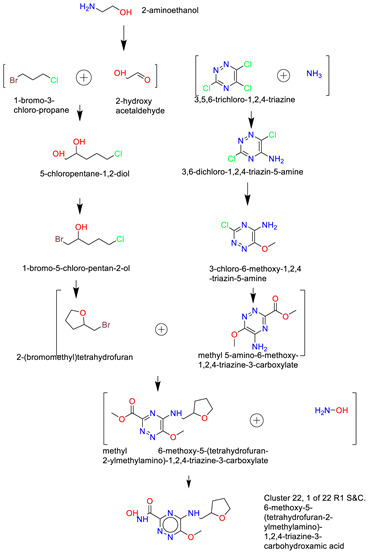

(2) **Cluster 10, 1 of 86**

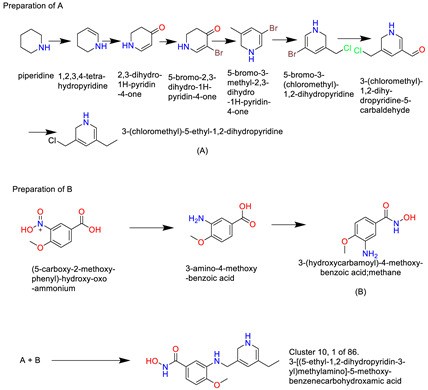

(3) **Cluster 3, 1 of 3**

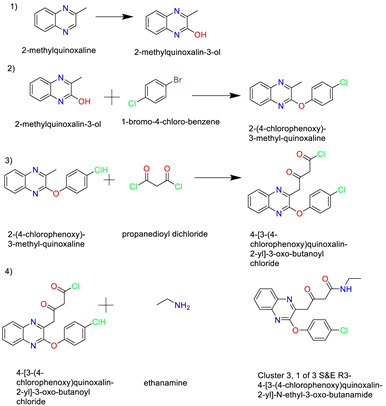

(4) **Cluster 16, 1 of 2**

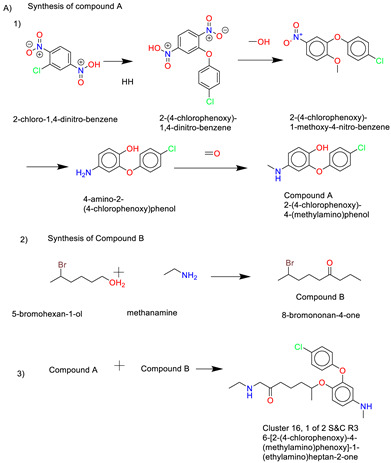

(5) **Cluster 8, 1 of 1**

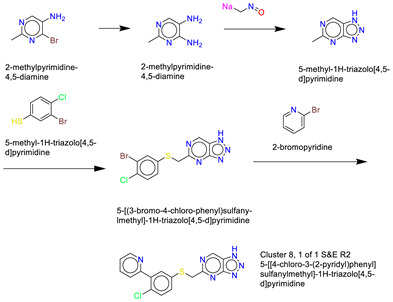

(6) **Cluster 8, 1 of 29**

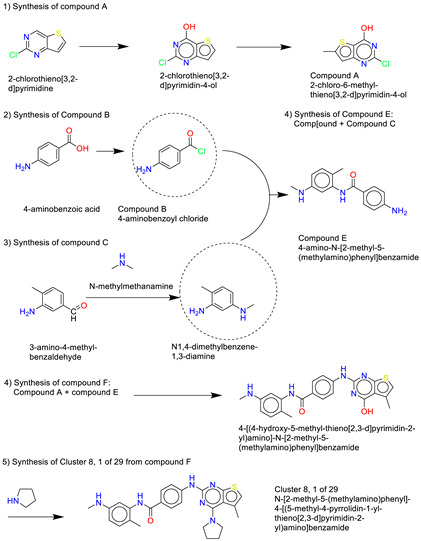

(7) **Cluster 25, 1 of 8**

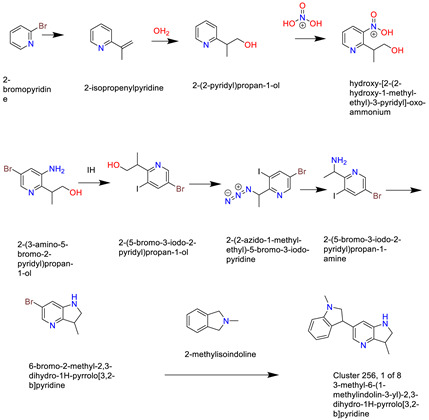

(8) **Cluster 12, 1 of 49**

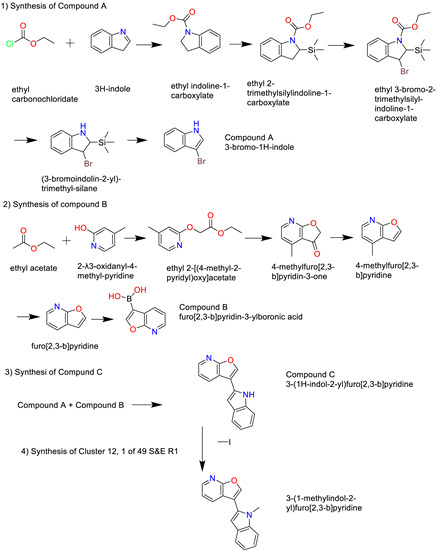

All data are available in the Supplementary Materials, see link below.

## 3. Discussion

The development of multi-target drugs is described as the process of “taking a well-validated primary target for a given disease and adding secondary activities to enhance efficacy” [37]. Designing such a drug involves fusing the inhibitory actions of two or more drugs into one molecule [38]. Typical approaches used for designing multi-target drugs include “Pharmacophore” and “Screening”. The pharmacophore approach can include processes such as merged-pharmacophore mode, fused-pharmacophore mode, non-cleavable linked pharmacophore, and cleavable pharmacophore. Pharmacophore modelling aims to “strip” functional groups of their true chemical nature in order to categorize them into a small number of pharmacophore types based on their predominant physicochemical characteristics [51]. Difficulties with this method occur due to inadequate or inaccurate conformational sampling, ambiguities in pharmacophore typing (primarily because of uncertainty regarding the tautomeric/protonation status of compounds), computer time limitations in complex molecular overlay calculations, and the selection of inappropriate anchoring points in active sites when ligand cocrystal structures are unavailable [51]. Along with pharmacophore the technique of screening is also used for drug discovery and has four identifiable categories: Fragment-based Drug Discovery (FBDD), High-throughput Screening (HTS), High-content Screening (HCS), and Virtual Screening (VS). For the work described here, a modified version of the Virtual Screening (VS) programme is used to enable the discovery of multi-target compounds (inhibitors). Using computational methods available to medicinal chemistry, computer-aided drug design and molecular modelling have greatly assisted drug design in the field [11]. Computational methods such as molecular docking, homology modelling, molecular dynamics, and quantitative structure–activity relationships (QSAR) are frequently used as part of the process for finding new therapeutic drug targets using computational methods [12]. For this work, eight compounds (inhibitors) were identified as possible inhibitors for four targets: HDAC, BRD, HH, and TRK.

Having selected four targets used in the study of NBS4, two receptors from each of the four targets were selected and the similarities of the receptors were compared as representations of the targets (Figure 4). Results indicated 80–100% similarities (using the Protein Aligner programme from Samson) confirming the possibility for multi-target use. High similarity between receptors indicates their suitability for use in cross-docking and is a process that involves selecting known lead compounds to produce hitlists of compounds (clusters). Using BROOD [39] as part of the OpenEye suite, several BROOD rounds were completed on the lead compounds to produce hitlists with the top 25 of each hitlist selected to run on OEDocking [40], AFITT [42], ROCS [41], and VIDA. The hitlists of compounds (clusters) were docked and the selected compounds, cross-docked. The clusters were docked with more than one docking programme as a means to validating the result.

The final ranking of the selected clusters was performed on AFITT as the receptor preparation with the tool MakeReceptor gives the user more control over the receptor-creating process. AFITT also has the advantage of real-space fitting of ligands in density, integrated with REFMAC, PHENIX, BUSTER, CNX, and COOT, and also fragment and cocktail fitting. In AFFIT, it is possible to select more than one ligand to fit generation of high-quality refinement dictionaries for use. This can be performed during reciprocal space refinement that includes the following: the use of forcefield (MMFF); semi-empirical (AM1, PM3) methods during reciprocal space refinement for BUSTER and Phenix; real space fitting of protein residues; proper handling of covalently bonded ligands, and proper handling of multiple occupancy ligands.

The ranking is based not only on the best results but also on the ability of the identified compounds to cross-dock on the receptors of other targets, and it was this process that led to the selection of the eight compounds. Using the tool ROCS provided cluster validation and is based on a large database search. Toxicity and mutagenicity of the eight compounds were tested using Toxicity Estimation Software Tools (TEST) [48], and the results showed some similarity with the original lead compounds (Table 7). With the aid of the retro-synthesis programme Spaya (IKTOS), the possibility of synthesizing all eight compounds was demonstrated (see the Results section) [49]. These results point the way to future work that will focus on preparing and testing the eight compounds in vitro and in vivo [52].

## 4. Materials and Methods

### 4.1. Materials

Computer programmes:

1. OpenEye Scientific programmes, which include various applications, were used. The suite comprises BROOD, MakeReceptor, FRED, and AFITT.
2. Molegro Virtual Docker.
3. The Samson suite includes Autodock Vina Extended, the Fitted suite by Molecular Forecaster, and Protein Aligner.
4. Toxicity Estimation Software Tools (TEST).
5. BIOVIA Discovery Studio Visualizer.
6. Spaya retrosynthesis software.

### 4.2. Method

1. Identifying drug targets.
2. Selection of two proteins (receptors) for each target and downloading the PDB files and their electron density map from the Protein Data Bank database.
3. Comparing the binding/active sites similarities of the receptors. Run protein similarity on Samson (Protein Aligner) to determine suitability.
4. Selection of two lead compounds from each type.
5. Run the lead compounds on BROOD (from the OpenEye suite) and produce hit lists using Shape and Colour and Shape and Electrostatics.
6. Receptor preparations using MakeReceptor from the OpenEye suite.
7. Docking the hit compounds with OpenEye suite (FRED), Molegro, and Samson suite (AutoDockVina and Fitted).
8. Run cross-docking; each hitlist clusters from one target to the other 3 targets (using their protein/receptor).
9. Run hits with AFITT to rank the compounds according to their fitting probabilities.
10. Run selected clusters on ROCS.
11. Run selected clusters on Toxicity Estimation Software Tools (TEST)
12. Run clusters on Spaya to find the best synthesis route.

## 5. Conclusions

Using Virtual Screening with some modifications, eight compounds were identified as potential inhibitors across four targets (HDAC, BRD, HH, TRK) for the development of multi-target drug treatment of NBS4. The next stage is for the eight compounds to undergo single molecule testing in vivo and in vitro [52].

## Figures and Tables

**Table 2:** Summary of the rounds on BROOD for generating hitlists for each target. The top 25 compounds were selected from each round.

Target	Shape and Colour	Shape and Electrostatics
1- Histone Deacetylase 8 Inhibitors (8b and 20a)	8b-2 rounds 20a-2 rounds	8b-2 rounds 20a-2 rounds
2- Bromodomain Inhibitors (JQ1 and I-BET762)	JQ1-2 rounds I-BET-762-3 rounds	JQ1-2 rounds I-BET-762-3 rounds
3- HH inhibitors (BMS-833923 and Vismodegib)	BMS-833923-2 rounds Vismodegib-2 rounds	BMS-833923-2 rounds Vismodegib-2 rounds
4- Tropomyosin Receptor Kinase Inhibitors (GW441759 and 10)	GW441759-2 rounds Compound **10**-2 rounds	GW441759-2 rounds Compound **10**-2 rounds

**Table 3:** Selected clusters from each target (total n = 35).

HDACIs (n = 9)	BRDI (n = 8)	HH (n = 10)	TRK (n = 8)
Cluster 22, 1 of 22	Cluster 3, 1 of 3	Cluster 4, 1 of 1	Cluster 12, 1 of 6
Cluster 21, 1 of 11	Cluster 25, 1 of 12	Cluster 8, 1 of 1	Cluster 4, 1 of 5
Cluster 16,1 of 65	Cluster 16, 1 of 2	Cluster 1, 1 of 3	Cluster 8, 1 of 19
Cluster 23, 1 of 1	Cluster 15, 1 of 2	Cluster 9, 1 of 1	Cluster 9, 1 of 4
Cluster 1, 1 of 26	Cluster 4, 1 of 4	Cluster 8, 1 of 29	Cluster 25, 1 of 8
Cluster 7, 1 of 26	Cluster 24, 1 of 7	Cluster 21, 1 of 99	Cluster 12, 1 of 49
Cluster 4, 1 of 28	Cluster 23, 1 of 1	Cluster 15, 1 of 6	Cluster 7, 1 of 11
Cluster 10, 1 of 86	Cluster 10, 1 of 1	Cluster 23, 1 of 1	Cluster 25, 1 of 8
Cluster 12, 1 of 50		Cluster 17, 1 of 1	
		Cluster 20, 1 of 12	

**Table 4:** The top clusters obtained from BROOD BRD receptor 4BJX were docked with AFITT, FRED, AutoDock Vina Extended, Molegro, and Fitted. BET762 and JQ1 were the lead compounds.

Column 1	Clusters from Bromodomain (BRD)	AFITT	FRED	AutoDock Vina Extended	Molegro	Fitted
1	Cluster 3, 1 of 3	0.766	−6.474	−8.124	−4.86	−25.259
2	Cluster 25, 1 of 12	0.6863	−8.315	−8.549	89.3	−26.775
3	Cluster 16, 1 of 2	0.7356	−6.337	−7.542	52.9	−21.687
4	Cluster 15, 1 of 2	0.553	−5.308	−7.048	33.28	−23.624
5	Cluster 4, 1 of 4	0.4898	−5.693	−8.403	36.98	−25.566
6	Cluster 24, 1 of 7	0.4644	−8.556	−8.909	80.89	−30.289
7	Cluster 23, 1 of 1	0.497	−5.955	−7.795	9.34	−24.131
8	Cluster 10, 1 of 1	0.7103	−4.672	−7.71	5.23	−21.734
9	BET-762-Lead compound	0.6691	−7.528	−7.971	49.84	−19.709
10	JQ1-Lead compound	0.6084	−8.133	−7.006	99.29	−22.161

**Table 5:** Docking of selected clusters from each target with more than one receptor of the same target. Data represent the best real space correlation coefficient calculation (RSCC).

Clusters HDACs	AFITT		Clusters BRD	AFITT		Clusters HH	AFITT		Clusters Tropomyosin	AFITT	
	2V5X	2V5W		4BJX	5UY9		5L7I	3N1P		4AT3	3V5Q
Cluster 22, 1 of 22	0.652	0.541	Cluster 3, 1 of 3	0.77	0.42	Cluster 4, 1 of 1	0.552	0.336	Cluster 12, 1 of 6	0.638	0.438
Cluster 21, 1 of 11	0.650	0.499	Cluster 25, 1 of 12	0.69	0.39	Cluster 8, 1 of 1	0.514	0.385	Cluster 4, 1 of 5	0.685	0.438
Cluster 16,1 of 65	0.650	0.541	Cluster 16, 1 of 2	0.74	0.33	Cluster 1, 1 of 3	0.521	0.330	Cluster 8, 1 of 19	0.435	0.438
Cluster 23, 1 of 1	0.645	0.532	Cluster 15, 1 of 2	0.55	0.39	Cluster 9, 1 of 1	0.513	0.404	Cluster 9, 1 of 4	0.622	0.539
Cluster 1, 1 of 26	0.642	0.504	Cluster 4, 1 of 4	0.49	0.33	Cluster 8, 1 of 29	0.496	0.432	Cluster 25, 1 of 8	0.675	0.521
Cluster 7, 1 of 26	0.635	0.527	Cluster 24, 1 of 7	0.46	0.37	Cluster 21, 1 of 99	0.493	0.340	Cluster 12, 1 of 49	0.630	0.627
Cluster 4, 1 of 28	0.633	0.499	Cluster 23, 1 of 1	0.50	0.37	Cluster 15, 1 of 6	0.491	0.374	Cluster 7, 1 of 11	0.643	0.596
Cluster 10, 1 of 86	0.632	0.517	Cluster 10, 1 of 1	0.71	0.37	Cluster 23, 1 of 1	0.478	0.470	Cluster 25, 1 of 11	0.491	0.614
Cluster 12, 1 of 50	0.631	0.524	BET-762	0.67	0.39	Cluster 17, 1 of 1	0.471	0.364	Compound **Z9**	0.596	0.543
20A	0.631	0.511	JQ1	0.61	0.41	Cluster 20, 1 of 12	0.463	0.389	Compound **10**	0.560	0.505
8B	0.629	0.516				Vesmodigib	0.363	0.363			
						BMS-833923		0.313			

**Table 6:** Selected clusters from each type that docked with the rest of the targets (n = 8).

HDACIs (n = 2)	BRDI (n = 2)	HH (n = 2)	TRK (n = 2)
Cluster 22, 1 of 22	Cluster 3, 1 of 3	Cluster 8, 1 of 1	Cluster 25, 1 of 8
Cluster 10, 1 of 86	Cluster 16, 1 of 2	Cluster 8, 1 of 29	Cluster 12, 1 of 49

**Table 7:** Results from the Toxicity Estimation Software Tools (TEST); predictive values.

Clusters	Bioconcentration Factor ^1^	Mutagenicity ^2^	Oral rat LD_50_ -Log10 (mol/kg) ^3^	T. Pyriformis IGC_50_ (48 h) mg/L ^4^
Cluster 22, 1 of 22	0.31	Positive	1.78	3845.78
Cluster 10, 1 of 86	5.56	Positive	2.67	173.52
Cluster 3, 1 of 3	12.71	Positive	2.52	4.61
Cluster 16, 1 of 2	46.62	Negative	2.66	1.91
Cluster 8, 1 of 1	27.88	Negative	1.70	6.33
Cluster 8, 1 of 29	8.62	N/A	2.52	N/A
Cluster 25, 1 of 8	98.26	Positive	2.01	6.55
Cluster 12, 1 of 49	308.45	Negative	2.41	6.46
20A	9.94	Positive	N/A	36.39
8B	5.14	Positive	N/A	29.50
BET-762	22.21	Negative	2.26	2.27
JQ1	235.52	Negative	2.45	0.73
Vesmodigib	28.48	Negative	2.13	2.87
BMS-833923	11.53	Positive	2.38	N/A
Compound **Z9**	25.14	Positive	2.20	42.96
Compound **10**	20.85	Positive	2.65	7.04

^1^ Bioconcentration factor: ratio of the chemical concentration in fish as a result of absorption via the respiratory surface to that in water at a steady state. ^2^ Ames mutagenicity: A compound is positive for mutagenicity if it induces revertant colony growth in any strain of Salmonella typhimurium. ^3^ Oral rat LD_50_: Amount of chemical (mg/kg body weight) that causes 50% of rats to die after oral ingestion. ^4^ 48 h T. pyriformis IGC_50_: Concentration of the test chemical in water (mg/L) that causes 50% growth inhibition to Tetrahymena pyriformis after 48 h.

## Data Availability

See the Supplementary Materials https://doi.org/10.5281/zenodo.14959257.

## Supplementary Materials

The following supporting information can be downloaded at: https://doi.org/10.5281/zenodo.14959257, accessed on 20 March 2025.

## References

[1] Cullinane C.J., Burchill S.A., Jeremy A.S., O’Leary J.J., Lewis I.J. (2003). Molecular Biology and Pathology of Paediatric Cancer.

[2] Paraboschi I., Privitera L., Kramer-Marek G., Anderson J., Giuliani S. (2021). Novel Treatments and Technologies Applied to the Cure of Neuroblastoma. Children.

[3] Cohen S., Carpenter G. (1975). Human epidermal growth factor: Isolation and chemical and biological properties. Proc. Natl. Acad. Sci. USA.

[4] Gerges A., Canning U. (2024). Neuroblastoma and its Target Therapies: A Medicinal Chemistry Review. ChemMedChem.

[5] Zafar A., Wang W., Liu G., Wang X., Xian W., McKeon F., Foster J., Zhou J., Zhang R. (2021). Molecular targeting therapies for neuroblastoma: Progress and challenges. Med. Res. Rev..

[6] Inomistova M., Khranovska N., Skachkova O. (2019). Role of Genetic and Epigenetic Alterations in Pathogenesis of Neuroblastoma.

[7] Bhoopathi P., Mannangatti P., Emdad L., Das S.K., Fisher P.B. (2021). The quest to develop an effective therapy for neuroblastoma. J. Cell Physiol..

[8] Zhang W.L., Pei J.F., Lai L.H. (2017). Computational Multitarget Drug Design. J. Chem. Inf. Model..

[9] Anighoro A., Bajorath J., Rastelli G. (2014). Polypharmacology: Challenges and Opportunities in Drug Discovery. J. Med. Chem..

[10] Knight Z.A., Lin H., Shokat K.M., Knight Z.A., Lin H., Shokat K.M. (2010). Targeting the cancer kinome through polypharmacology. Nat. Rev. Cancer.

[11] Ooms F. (2000). Molecular Modeling and Computer Aided Drug Design. Examples of their Applications in Medicinal Chemistry. Curr. Med. Chem..

[12] Kumalo H.M., Bhakat S., Soliman M.E.S., Kumalo H.M., Bhakat S., Soliman M.E.S. (2015). Theory and Applications of Covalent Docking in Drug Discovery: Merits and Pitfalls. Molecules.

[13] Bolden J.E., Peart M.J., Johnstone R.W. (2006). Anticancer activities of histone deacetylase inhibitors. Nat. Rev. Drug Discov..

[14] Oehme I., Deubzer H.E., Wegener D., Pickert D., Linke J.P., Hero B., Kopp-Schneider A., Westermann F., Ulrich S.M., Von Deimling A. (2009). Histone Deacetylase 8 in Neuroblastoma Tumorigenesis. Clin. Cancer Res..

[15] Heimburg T., Kolbinger F.R., Zeyen P., Ghazy E., Herp D., Schmidtkunz K., Melesina J., Shaik T.B., Erdmann F., Schmidt M. (2017). Structure-Based Design and Biological Characterization of Selective Histone Deacetylase 8 (HDAC8) Inhibitors with Anti-Neuroblastoma Activity. J. Med. Chem..

[16] Vannini A., Volpari C., Gallinari P., Jones P., Mattu M., Carfí A., Francesco R.D., Steinkühler C., Marco S.D. (2007). Substrate binding to histone deacetylases as shown by the crystal structure of the HDAC8–substrate complex. EMBO Rep..

[17] Tamkun J.W., Deuring R., Scott M.P., Kissinger M., Pattatucci A.M., Kaufman T.C., Kennison J.A. (1992). brahma: A regulator of Drosophila homeotic genes structurally related to the yeast transcriptional activator SNF2/SWI2. Cell.

[18] Pérez-Salvia M., Esteller M. (2017). Bromodomain inhibitors and cancer therapy: From structures to applications. Epigenetics.

[19] Li G.-Q., Guo W.-Z., Zhang Y., Seng J.-J., Zhang H.-P., Ma X.-X., Zhang G., Li J., Yan B., Tang H.-W. (2016). Suppression of BRD4 inhibits human hepatocellular carcinoma by repressing MYC and enhancing BIM expression. Oncotarget.

[20] McKeown M.R., Shaw D.L., Fu H., Liu S., Xu X., Marineau J.J., Huang Y., Zhang X., Buckley D.L., Kadam A. (2014). Biased multicomponent reactions to develop novel bromodomain inhibitors. J. Med. Chem..

[21] Mirguet O., Gosmini R., Toum J., Clement C.A., Barnathan M., Brusq J.M., Mordaunt J.E., Grimes R.M., Crowe M., Pineau O. (2013). Discovery of epigenetic regulator I-BET762: Lead optimization to afford a clinical candidate inhibitor of the BET bromodomains. J. Med. Chem..

[22] Wyce A., Ganji G., Smitheman K.N., Chung C.-w., Korenchuk S., Bai Y., Barbash O., Le B., Craggs P.D., McCabe M.T. (2013). BET Inhibition Silences Expression of MYCN and BCL2 and Induces Cytotoxicity in Neuroblastoma Tumor Models. PLoS ONE.

[23] Peukert S., Miller-Moslin K. (2010). Small-Molecule Inhibitors of the Hedgehog Signaling Pathway as Cancer Therapeutics. ChemMedChem.

[24] Nüsslein-volhard C., Wieschaus E. (1980). Mutations affecting segment number and polarity in Drosophila. Nature.

[25] Lum L., Beachy P.A. (2004). The Hedgehog Response Network: Sensors, Switches, and Routers. Science.

[26] Skoda A.M., Simovic D., Karin V., Kardum V., Vranic S., Serman L. (2018). The role of the Hedgehog signaling pathway in cancer: A comprehensive review. Bosn. J. Basic Med. Sci..

[27] Goodrich L.V., Scott M.P. (1998). Hedgehog and patched in neural development and disease. Neuron.

[28] Siu L.L., Papadopoulos K., Alberts S.R., Kirchoff-Ross R., Vakkalagadda B., Lang L., Ahlers C.M., Bennett K.L., Tornout J.M.V. (2010). A first-in-human, phase I study of an oral hedgehog (HH) pathway antagonist, BMS-833923 (XL139), in subjects with advanced or metastatic solid tumors. J. Clin. Oncol..

[29] Dlugosz A., Agrawal S., Kirkpatrick P. (2012). Vismodegib. Nat. Rev. Drug Discov..

[30] Deshpande I., Liang J., Hedeen D., Roberts K.J., Zhang Y., Ha B., Latorraca N.R., Faust B., Dror R.O., Beachy P.A. (2019). Smoothened stimulation by membrane sterols drives Hedgehog pathway activity. Nature.

[31] Kavran J.M., Ward M.D., Oladosu O.O., Mulepati S., Leahy D.J. (2010). All Mammalian Hedgehog Proteins Interact with Cell Adhesion Molecule, Down-regulated by Oncogenes (CDO) and Brother of CDO (BOC) in a Conserved Manner. J. Biol. Chem..

[32] McCarthy C., Walker E. (2014). Tropomyosin receptor kinase inhibitors: A patent update 2009–2013. Expert Opin. Ther. Pat..

[33] Bailey J.J., Jaworski C., Tung D., Wängler C., Wängler B., Schirrmacher R. (2020). Tropomyosin receptor kinase inhibitors: An updated patent review for 2016–2019. Expert Opin. Ther. Pat..

[34] Cocco E., Scaltriti M., Drilon A. (2018). NTRK fusion-positive cancers and TRK inhibitor therapy. Nat. Rev. Clin. Oncol..

[35] Saleh K., Khalifeh-Saleh N., Kourie H.R. (2019). TRK inhibitors: Toward an era of agnostic targeted therapies in oncology. Pharmacogenomics.

[36] Bernard-Gauthier V., Aliaga A., Aliaga A., Boudjemeline M., Hopewell R., Kostikov A., Rosa-Neto P., Thiel A., Schirrmacher R. (2015). Syntheses and evaluation of carbon-11- and fluorine-18-radiolabeled pan-tropomyosin receptor kinase (Trk) inhibitors: Exploration of the 4-aza-2-oxindole scaffold as Trk PET imaging agents. ACS Chem. Neurosci..

[37] Morphy R., Kay C., Rankovic Z. (2004). From magic bullets to designed multiple ligands. Drug Discov. Today.

[38] Li X., Li X., Liu F., Li S., Shi D. (2021). Rational Multitargeted Drug Design Strategy from the Perspective of a Medicinal Chemist. J. Med. Chem..

[39] Wang L.-h., Evers A., Monecke P., Naumann T. (2012). Ligand based lead generation—Considering chemical accessibility in rescaffolding approaches via BROOD. J. Cheminformatics.

[40] McGann M. (2012). FRED and HYBRID docking performance on standardized datasets. J. Comput.-Aided Mol. Des..

[41] López-Ramos M., Perruccio F. (2010). HPPD: Ligand-and Target-Based Virtual Screening on a Herbicide Target. J. Chem. Inf. Model.

[42] Janowski P.A., Moriarty N.W., Kelley B.P., Case D.A., York D.M., Adams P.D., Warren G.L. (2016). Improved ligand geometries in crystallographic refinement using AFITT in PHENIX. Acta Cryst..

[43] (2024). VIDA.

[44] Moitessier N., Pottel J., Therrien E., Englebienne P., Liu Z., Tomberg A., Corbeil C.R. (2016). Medicinal Chemistry Projects Requiring Imaginative Structure-Based Drug Design Methods. Acc. Chem. Res..

[45] Thomsen R., Christensen M.H. (2006). MolDock:  A New Technique for High-Accuracy Molecular Docking. J. Med. Chem..

[46] (2023). Discovery Studio Visualizer.

[47] Samson Software Protein Aligner. https://www.uniprot.org/align.

[48] Martin T., Harten P., Venkatapathy R., Young D. T.E.S.T., EPA-ORD-CCTE Clowder: Public Domain Dedication, 2021. https://www.epa.gov/comptox-tools/toxicity-estimation-software-tool-test.

[49] Parrot M., Tajmouati H., da Silva V.B.R., Atwood B.R., Fourcade R., Gaston-Mathé Y., Do Huu N., Perron Q., Parrot M., Tajmouati H. (2023). Integrating synthetic accessibility with AI-based generative drug design. J. Cheminformatics.

[50] OpenEye, Cadence Molecular Sciences (2023). BROOD.

[51] Horvath D. (2010). Pharmacophore-Based Virtual Screening. Methods Mol. Biol..

[52] Korshavn K.J., Jang M., Kwak Y.J., Kochi A., Vertuani S., Bhunia A., Manfredini S., Ramamoorthy A., Lim M.H. (2015). Reactivity of Metal-Free and Metal-Associated Amyloid-beta with Glycosylated Polyphenols and Their Esterified Derivatives. Sci. Rep..

